# Immunophenotypic features of tumor infiltrating lymphocytes from mammary carcinomas in female dogs associated with prognostic factors and survival rates

**DOI:** 10.1186/1471-2407-10-256

**Published:** 2010-06-04

**Authors:** Alessandra Estrela-Lima, Márcio SS Araújo, João M Costa-Neto, Andréa Teixeira-Carvalho, Stella M Barrouin-Melo, Sergio V Cardoso, Olindo A Martins-Filho, Rogéria Serakides, Geovanni D Cassali

**Affiliations:** 1 Departamento de Patologia e Clínicas, Escola de Medicina Veterinária-Universidade Federal da Bahia, Salvador, Bahia, Brasil; 2 Laboratório de Biomarcadores de Diagnóstico e Monitoração-Centro de Pesquisas René Rachou-Fundação Oswaldo Cruz, Belo Horizonte, Minas Gerais, Brasil; 3 Área de Patologia, Faculdade de Odontologia - Universidade Federal de Uberlândia, Minas Gerais, Brasil; 4 Departamento de Clinicas e Cirurgia, Escola de Veterinária-Universidade Federal de Minas Gerais, Belo Horizonte, Minas Gerais, Brasil; 5 Laboratório de Patologia Comparada (LPC), Departamento de Patologia Geral, Instituto de Ciências Biológicas-Universidade Federal de Minas Gerais, Belo Horizonte, Minas Gerais, Brasil

## Abstract

**Background:**

The immune system plays an important role in the multifactorial biologic system during the development of neoplasias. However, the involvement of the inflammatory response in the promotion/control of malignant cells is still controversial, and the cell subsets and the mechanisms involved are poorly investigated. The goal of this study was to characterize the clinical-pathological status and the immunophenotyping profile of tumor infiltrating lymphocytes and their association with the animal survival rates in canine mammary carcinomas.

**Methods:**

Fifty-one animals with mammary carcinomas, classified as carcinomas in mixed tumors-MC-BMT = 31 and carcinomas-MC = 20 were submitted to systematic clinical-pathological analysis (tumor size; presence of lymph node and pulmonary metastasis; clinical stage; histological grade; inflammatory distribution and intensity as well as the lymphocytic infiltrate intensity) and survival rates. Twenty-four animals (MC-BMT = 16 and MC = 8) were elected to the immunophenotypic study performed by flow cytometry.

**Results:**

Data analysis demonstrated that clinical stage II-IV and histological grade was I more frequent in MC-BMT as compared to MC. Univariate analysis demonstrated that the intensity of inflammation (moderate/intense) and the proportion of CD4^+ ^(≥ 66.7%) or CD8^+ ^T-cells (<33.3%) were not associated with worse survival rate. Multivariate analysis demonstrated that only lymphocytic infiltrate intensity ≥ 600 (*P *= 0.02) remained as independent prognostic factor. Despite the clinical manifestation, the lymphocytes represented the predominant cell type in the tumor infiltrate. The percentage of T-cells was higher in animals with MC-BMT without metastasis, while the percentage of B-lymphocytes was greater in animals with metastasized MC-BMT (*P *< 0.05). The relative percentage of CD4^+ ^T-cells was significantly greater in metastasized tumors (both MC-BMT and MC), (*P *< 0.05) while the proportion of CD8^+ ^T-cells was higher in MC-BMT without metastasis. Consequently, the CD4^+^/CD8^+ ^ratio was significantly increased in both groups with metastasis. Regardless of the tumor type, the animals with high proportions of CD4^+ ^and low CD8^+ ^T-cells had decreased survival rates.

**Conclusion:**

The intensity of lymphocytic infiltrate and probably the relative abundance of the CD4^+ ^and CD8^+ ^T-lymphocytes may represent important survival prognostic biomarkers for canine mammary carcinomas.

## Background

Spontaneous mammary tumors of female dogs have various epidemiological, clinical, biological and genetic characteristics that are similar to those in women [1]. Several researchers have proposed the use of these tumors as models for comparative studies with humans [2-6].

The development of malignant tumors is controlled by a multifactorial biologic system that depends on genetic abnormalities as well as the interplay between tumor cells, stromal cells, and host inflammatory cells [7]. The inflammatory process associated with neoplastic growth involves a complex host response [8-10]. This response includes the innate immune response, and the highly specific but more slowly developing adaptive or acquired immune response, mediators and the interactions between chemokines, cytokines and receptors [11-13].

In certain situations, the cells responsible for modulation of the inflammatory response release chemokines and cytokines that stimulate cellular proliferation and angiogenesis, as well as inhibiting apoptosis, thus altering the immune response to aggression [8,14,15]. There is evidence that major inflammatory cytokines (such as IL-1β, IL-6, IL-23 and TNF-α promote tumor development by acting directly or indirectly on neoplastic cells [10,15,16]. These factors together can accelerate mutagenesis and promote the survival of atypical clones with a greater capacity to invade tissues and organs [12].

Histological and immunohistochemical studies have demonstrated that mammary carcinomas are effectively infiltrated by different types of leukocytes, predominantly macrophages and lymphocytes consisting mainly of T-cells [17-19]. Initially, it was postulated that the presence of the inflammatory infiltrate in the tumor site was evidence of immune activity against the neoplastic growth. The functional role of tumor-infiltrating lymphocytes in dogs with mammary tumors is not yet fully established [20].

Thus, there is no consensus regarding the effectiveness of the anti-tumor response mediated by lymphocytes, or by the predominant lymphocyte subpopulations [7,18-22]. Nowak et al. (2007) [23] reported a correlation between the number of CD8^+ ^cells and the metastatic potential of mammary adenocarcinomas in female dogs. However, the inflammatory response associated with mammary carcinomas and its relationship with prognostic factors and the survival rate of female dogs has not been studied.

Aiming to understand the dynamic interaction and the association between host immune response and tumor development, this study was to evaluate the immunophenotypic features of infiltrating lymphocytes in canine mammary carcinomas and their relation to prognostic factors and survival.

## Methods

### Groups of animals

Fifty-one female dogs with mammary neoplasias measuring three centimeters or greater admitted at the Veterinarian Hospital of the *Universidade Federal da Bahia *(UFBA) were selected between October, 2008 to August, 2009. The animals were purebreds or mixed-breed with age ranging from of 8-18 years.

The tumor samples were classified based on the histopathological diagnosis according to Misdorp *et al*. (1999) [24] complemented by the proposal of Cassali et al (2002) for classification of micropapillary carcinoma [25] and the animals divided into two groups: i) carcinomas in benign mixed tumors (MC-BMT, n = 31) and ii) carcinomas (MC, n = 20). Histological data revaluated that MC group comprised distinct subtypes, including tubular (n = 3), papillary (n = 4), tubular-papillary (n = 3), solid (n = 4), micropapillary (n = 3), anaplastic (n = 2) and one special type referred as mucinous carcinoma, characterized by abundant mucin production. The study series of carcinomas was a selected series consisting as simple and mixed carcinomas exclusively.

Further categorization based on histological analysis of lymph node was further used to categorize the tumor samples into two subgroups referred as: i) (-) without lymph node metastasis [MC-BMT (-), n = 19; MC (-), n = 10] and ii) (+) with lymph node metastasis [MC-BMT (+), n = 12; MC (+), n = 10].

The clinical-pathological parameters, including tumor size, lymph nodes metastasis, pulmonary metastasis, clinical stage, histological grade, inflammation distribution and inflammation intensity, were used during comparative analysis between MC-BMT and MC.

Forty six animals were elected for the survival analysis [MC-BMT (-), n = 18; MC-BMT (+), n = 12; MC (-), n = 09; MC (+), n = 07]. Five out of 51 animals were excluded from the survival analysis because the owners have decided for euthanasia of the animals due to the advanced stage of the disease, the weakness of the animals or the impossibility of surgical excision, which was certified by the clinical, laboratory and radiological evaluations.

Twenty-four tumors samples were eligible for immunophenotypic analysis, yielding at least 2 × 10^6 ^leucocytes, including sixteen samples from the MC-BMT [MC-BMT (-), n = 08; MC-BMT (+), n = 08] and eight from the MC group [MC (-), n = 05; MC (+), n = 03].

After euthanasia, the diagnosis was confirmed by complete necropsy and histopathological analysis. None of the female dogs had prior use of anti-neoplastic drugs, anti-inflammatory drugs or antibiotics within the 30 days prior to surgical excision.

All procedures in this study were according to the guidelines set by the *Colégio Brasileiro de Experimentação Animal *(COBEA). This study was approved by the Ethical Committee for the use of Experimental Animals of the *Universidade Federal de Minas Gerais*, Brazil (CETEA).

### Clinical staging

The female dogs were initially submitted to a systematic clinical examination along with macroscopic evaluation of tumor and lymph nodes, histopathology of regional lymph nodes and thoracic radiological examinations, in order to accomplish a complete staging categorization. Stage is determined from information on the size tumor (T), involvement of regional lymph node (N) and the presence or absence of distant metastases (M). Stages # II, III, IV and V were defined based on TNM system previously describe by Owen, 1980 [26].

The clinical anamnesis was carried out by detailed evaluation of physiological parameters, analysis of historical evolution and reproductive records along with biochemical and hematological analysis. The macroscopic evaluation included determination of tumor features (size, presence of inflammatory reaction and/or ulceration) and location and analysis of regional lymph nodes by palpation. The confirmation of neoplastic involvement was determined by histopathological analysis of inguinal and axillaries lymph nodes. Thoracic radiological examinations were performed in three planes (right lateral and left lateral and ventral-dorsal) to search for lung metastasis.

Upon suspect clinical diagnosis, forty-six female dogs underwent surgical excision by mastectomy (regional or radical depending on the tumor size, location and aspects of the lymphatic drainage). Immediately after surgery, the mammary chain was sent to the Department of Pathology, at *Universidade Federal da Bahia*, for macroscopic and microscopic characterization of the tumor features.

The forty six animals included in the survival rate analysis were submitted to quarterly follow-ups during twelve months, including systematic clinical evaluation, radiological examinations along with biochemical and hematological analysis. Survival rates were expressed in days between the surgical excisions of the end of follow-up.

### Tumor samples

The tumors were first classified according to size into two groups: i) tumors measuring between 3-5 centimeters and ii) tumors measuring more than 5 centimeters. In cases with multiple nodules, the larger tumor size was considered during the classification procedures. Following classification, depending on the tumor size, three or five fragments, representative of intratumor and peripheral tumor areas were randomly selected and removed from each tumor. Necrotic areas were excluded. Tumor fragments, measuring 1.5 × 1.5 cm, were submitted to histological, morphological/morphometric and immunophenotypic analysis.

### Histological classification and grade

Fragments of the affected mammary gland that included skin and subcutaneous tissues were fixed in phosphate buffered 10% neutral formalin and processed by the routine technique of paraffin embedding. Histological sections (4 μm) underwent hematoxylin-eosin (HE) staining [27]. In all cases, duplicate slides were prepared and analyzed by two veterinarian pathologists.

**Figure 1:**
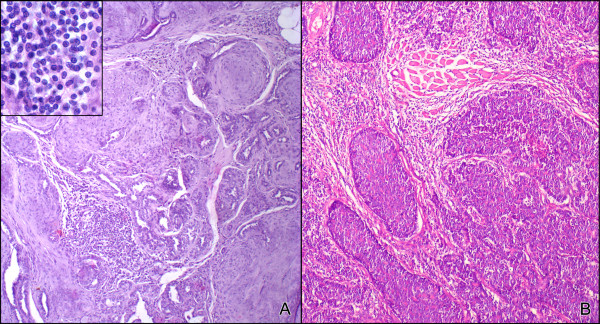
**Histological diagnosis of canine mammary carcinoma samples**. Low-power view of tumor specimen classified as carcinoma in benign mixed tumor (MC-BMT) associated with multifocal lymphocytic infiltrate, 200× (A); Higher power view of infiltrating lymphocytes in MC-BMT 600× (A-Inset); Low-power view of tumor specimen classified as mammary carcinoma (MC) in solid type associated with diffuse lymphocytic infiltrate, 200× (B). Histological sections (4 μm) underwent hematoxylin-eosin (HE) staining.

The histological classification was performed according to the World Health Organization system (WHO) [24] complemented by the proposal of Cassali et al (2002) for classification of micropapillary carcinoma [25]. Tumors with foci of malignant-appearing cells or distinct nodules of such cell occurring in complex adenomas or benign mixed tumors were diagnosed as carcinomas in benign mixed tumors (MC-BMT). Tumors composed of one type of malign cell either resembling luminal epithelial or myoepithelial cells were diagnosed as carcinomas (MC) [24]. Figure 1 shows representative images of MC-BMT (Figure 1A) and MC in solid growth pattern (Figure 1B).

Histological grade was defined according to Elston and Ellis (1991) [28] using 4 μm HE stained tissue sections. For nuclear pleomorphism, the score #1 was used when the nuclei were small, with little increase in size in comparison with normal breast epithelial cells, and had regular outlines and a uniformity of nuclear chromatin. The score of #2 was assigned when the cells were larger than normal, had open vesicular nuclei with visible nucleoli, and showed moderate variability in size and shape. The score #3 corresponded to marked variation in size and shape, especially when very large and bizarre nuclei were vesicular with prominent and often multiple nucleoli. Strict criteria for identification of mitotic figures were employed according to Diest et al. (1992) [29]. Mitotic activity was assessed as the number of mitosis cells per 10 fields, performed by two independent analysts in a blinded fashion, using an Olympus BX-40 microscope fitted to a 10× eyepiece and a 40× objective. Using this equipment, one high power field visualizes an area of 0.239 mm^2 ^[30]. Tumor up to 7 mitotic figures was scored as 1 point, 8-16 mitotic figures as 2 points and more than 17 mitotic figures as 3 points. Final histological grade was obtained by adding up the scores #1, #2 and #3 and classified as follows: i) grade I: 3-5 points, well differentiated; ii) grade II: 6-7 points, moderately differentiated and grade III: 8-9 points, poorly differentiated.

### Morphological/morphometric analysis of tumor inflammatory infiltrate

The analysis of morphological aspects was carried out in 4 μm HE stained histological sections and the inflammatory reaction classified by the distribution and by the intensity. The distribution of inflammatory infiltrates were evaluated in peripheral and intra-tumor areas and classified as: i) focal: presence of 1-3 inflammatory foci; ii) multifocal: presence of more than 3 inflammatory foci and iii) diffuse: presence of inflammatory cells evenly distributed in the tumor section. The intensity of the inflammatory reaction was categorized into three subgroups (discrete, moderate or intense) based on morphometric analysis of total inflammatory infiltrate.

The morphometric analysis was carried out in eight selected "Hot Spots" histological fields using an Olympus BX-40 microscope fitted to a 10× eyepiece and a 40× objective, representative of both peripheral and intra-tumor areas. Morphometric analysis was assessed as the number inflammatory cells per eight fields. Images were captured using an oil immersion 100× objective (a digital camera was adapted to an Olympus BX-40 microscope), and the capture software SPOT version 3.4.5. The inflammatory cells were characterized by image analysis (Corel Draw software version 7.468). Neutrophils, macrophages, lymphocytes and plasma cells were identified based on their morphological features and quantified in HE stained sections. The eosinophils were identified and quantified using additional analysis of Chromotrope 2R staining of serial histological sections [31]. The total number of cells was obtained by adding the eight fields analyzed. The inflammatory infiltrate was first classified as: i) discrete: presence of less than 500 inflammatory cells; iii) moderate: presence of 500-1000 inflammatory cells and iii) intense: presence of more than 1000 inflammatory cells. The cut-off edges were determined based on the mean number of cells. For data analysis, two intervals were used considering the intensity of the lymphocytic infiltrate: i) discrete + moderate (x< 600 lymphocytes) and ii) intense (≥ 600 lymphocytes), respectively.

### Immunophenotyping of tumor lymphocytic infiltrate by flow cytometry

Tumor fragments from twenty four dogs females, representative of peri/intra-tumor areas were available for immunophenotypic analysis by flow cytometry as suggested by Teixeira-Carvalho et al., 2002 [32], modified as follows: tissue fragments were immersed in cold RPMI 1640 (5 mL) in Petri dish and placed on ice for maceration. The tissue were processed in tissue grinder and then filtered on stainless steel gaze to obtain single cell suspension. The cell suspension was washed twice in RPMI-1640 and ressuspended at a concentration of 1 × 10^7^cells/mL. Aliquots of 40 μL (4 × 10^5 ^cells) were incubated for 30 minutes at room temperature in the dark in 12 × 75 mm polystyrene tubes with 10 μL of previously diluted monoclonal antibodies (mAbs), including: anti-canine CD4-FITC or RPE 1:320 (rat-IgG2a, clone YKIX302.9), anti-canine CD8-FITC or RPE 1:40 (rat-IgG1, clone YCATE55.9) and anti-canine B cell-RPE 1:160 (Mouse IgG1, Clone: CA2.1D6), all of which were produced by Serotec (Oxford, UK). The combinations and dilutions of mAbs were used as proposed by Araújo et al. (2008) [33]. After incubation, 1 mL of lysing solution (FACS brand lysing solution, Becton Dickinson, San Diego, CA, USA) was added to each tube while vortexing and incubated for 10 minute at room temperature in the dark. The negative controls used were mAbs of the same isotype, produced in the same species, and obtained from the same manufacturer. Stained cells were washed twice with 2 mL PBS (phosphate buffered saline 0.15 M, pH 7.2) and then fixed with 100 μL of FACS FIX solution (10.0 g/l paraformaldehyde, 10.2 g/l sodium cacodylate and 6.65 g/l sodium chloride). A total of 10,000 events were run in a FACSCalibur flow cytometer, using the Cell Quest™software (Becton Dickinson San Jose, CA, USA). Data analysis was performed by first gating the lymphocyte population based on their forward scatter (FSC) versus side scatter (SSC) properties. Following, the immunophenotypic features were analyzed on dual color FL1/FITC versus FL2/R-PE dot plots. The results were expressed as percentage of positive cells (T-cells and B cells) within gated lymphocytes and also as the relative percentage of CD4^+^, CD8^+ ^T-cells and the CD4^+^/CD8^+ ^T-cell ratio within gated T-cells. In the survival analysis two intervals were considered for the relative percentages of CD4^+ ^and CD8^+ ^T-cell subsets within gated T-cells: i) low (<66.7% of CD4^+ ^T-cells and ≥ 33.3% of CD8^+ ^T-cells) and ii) high (≥ 66.7% of CD4^+ ^T-cells and <33.3% of CD8^+ ^T-cells).

### Statistical methods

Fisher's exact test was performed to verify the significance of presumptive associations between categorical variables. Data were grouped as follows: histological diagnosis (MC-BMT or MC); tumor size (3-5 cm or >5 cm); lymph node metastasis (no or yes); pulmonary metastasis (no or yes); clinical stage (II, III, IV or V); histological grade (I, II or III); inflammatory distribution (focal, multifocal or diffuse) and inflammatory intensity (discrete, moderate or intense), lymphocytic infiltrate intensity (<600 or ≥ 600 lymphocytes) as well as the relative percentage of CD4^+ ^T-cells (≥ 66.7%) and CD8^+ ^T-cells (>33.3%).

For quantitative variables, Kolmogorov-Smirnov test initially assessed the normality of distribution. Then, differences between groups were assessed by Student's *t */Mann-Whitney's *U *tests (when two groups were compared), or ANOVA/Kruskal-Wallis test (more than two groups) whether parametric/non parametric samples were evaluated, respectively. In the same way, possible correlations were investigated by Pearson/Spearman tests.

Survival curves were generated by Kaplan-Meier estimation method and compared by Log-rank (Mantel-Cox) or Cox proportional hazards tests in univariate or multivariate analysis, respectively. Groups were determined as above.

In all instances, *p *value < 0.05 was considered to be statistically significant. Analyses were performed using the softwares Prism 5.0 (GraphPad, San Diego, CA, USA) or SPSS 17 (SPSS Inc., Chicago, IL, USA).

## Results

### Clinical and pathologic features

Comparative analysis of clinical and pathologic findings between MC-BMT and MC are presented in Table 1. MC was significantly associated with more frequent pulmonary metastasis, advanced clinical stage and higher histological grade.

**Table 1:** Pathological and clinical status of mammary carcinomas in female dogs

**Parameters**	**Total** **(n = 51)**	**MC-BMT** **(n = 31)**	**MC** **(n = 20)**
			
**Lymph node metastasis**	43.0% (22/51)	39.0% (12/31)	50.0% (10/20)
**Pulmonary metastasis**	13.7% (07/51)	9.7% (03/31)	20.0% (04/20)
**Histologic grade**			
***Grade I***	58.8% (30/51)	80.6% (25/31)	25.0% (05/20)
***Grade II***	29.4% (15/51)	19.0% (06/31)	45.0% (09/20)
***Grade III***	11.8% (06/51)	0.0% (0/31)	30.0% (06/20)
**Size**			
***3-5 cm***	35.3% (18/51)	35.5% (11/31)	35.0% (07/20)
***>5 cm***	64.7% (33/51)	64.5% (20/31)	65.0% (13/20)
**Clinical Staging**			
***Stage II***	29.4% (15/51)	29.0% (09/31)	30.0% (06/20)
***Stage III***	25.5% (13/51)	29.0% (09/31)	20.0% (04/20)
***Stage IV***	29.4% (15/51)	38.7% (12/31)	15.0% (03/20)
***Stage V***	15.7% (08/51)	3.2% (01/31)*	35.0% (07/20)

* Significant intragroup and intergroup difference at *P *< 0.05.

### Morphologic and morphometric analysis

Inflammation was more frequently diffuse and intense in MC than MC-BMT, but this association lack statistical significance, as shown in Table 1.

**Figure 2:**
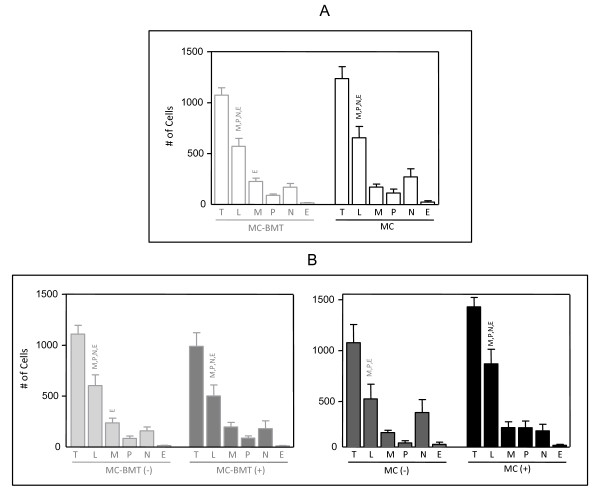
**Composition of inflammatory infiltrate associated with canine mammary carcinoma**. Infiltrating leukocyte populations from MC-BMT or MC (A), further subcategorized according to the absence (-) or presence (+) of lymph node metastasis (-) (B). The inflammatory cells were characterized by image analysis as described in Material and Methods. Data were expressed as number of cells (#) per eight "Hot Spots" histological fields. Significant differences at p < 0.05 are highlighted by asterisk.

Additional morphometry analysis revealed that lymphocytes were the predominant cell type, with significantly higher number in comparison to the other inflammatory cells (p < 0.001), except in the MC (-) subgroup, where the number of lymphocytes and neutrophils were similar (Figure. 2). No significant differences in the number or percentage of lymphocytes were observed between MC-BMT and MC groups (Figure. 2).

Further comparison between peripheral and intra-tumor areas did not demonstrated any significant differences in the morphological and morphometric features of the inflammatory infiltrates (data not shown).

### Flow cytometric immunophenotyping of tumor infiltrating lymphocytes

**Figure 3:**
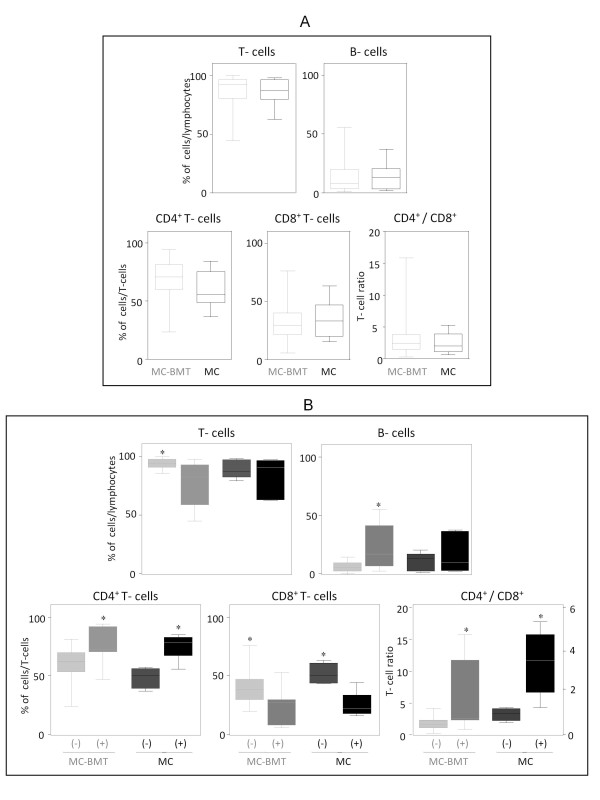
**Immunophenotypic profile of tumor infiltrating lymphocyte in canine mammary carcinomas**. Analysis of tumor infiltrating T-cells, B-lymphocytes and T-cell subsets from MC-BMT or MC (A), further subcategorized according to the absence (-) or presence (+) of lymph node metastasis (-) (B). Lymphocyte populations and subsets were identified by flow cytometric immunostaining as described in Material and Methods. Data were expressed as percentage of positive cells within gated lymphocytes and CD4^+^/CD8^+ ^T-cell ratio. Significant differences at p < 0.05 are highlighted by asterisk.

In this study we have evaluated 24 tumors, 16 from the MC-BMT and 8 from the MC group. The frequency of T and B-lymphocytes as well as CD4^+ ^and CD8^+ ^T-cell subsets and their ratio are shown in the Figure. 3A. Despite no significant differences were observed between the MC-BMT and MC groups, significant differences were observed when comparing subgroups of animals categorized based on the presence or absence of metastasis (Figure. 3B).

**Figure 4:**
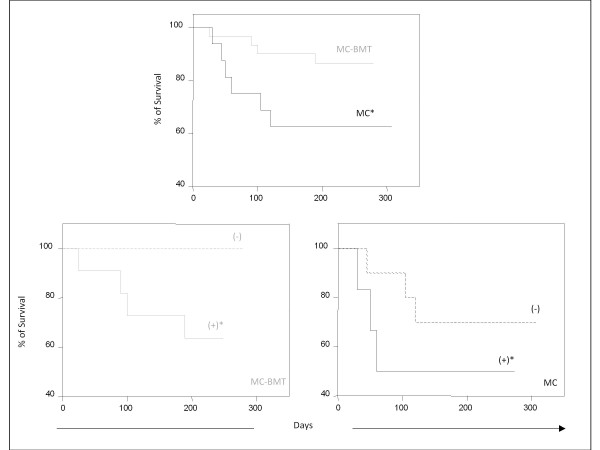
**Survival rates of animals with canine mammary carcinoma**. Kaplan-Meier survival curves for animals from MC-BMT or MC (top panel), further subcategorized according to the absence (-) or presence (+) of lymph node metastasis (-) (bottom panels). Animals were submitted to quarterly follow-ups during twelve months and survival rates (%) expressed in days between the surgical excisions of the end of follow-up as described in Methods. The survival curves were estimated with the Kaplan-Meier method followed by Log-rank test and significant differences at p < 0.05 highlighted by asterisk.

Data analysis demonstrate that MC-BMT(-) had significantly higher (*P *< 0.05) percentage of T-cells in comparison to MC-BMT(+). On the other hand, significant (*P *< 0.05) higher percentage of B-lymphocytes were observed in MC-BMT(+) in comparison to MC-BMT(-) (Figure. 4). There were no significant differences in the percentage of T and B-lymphocytes between the MC subgroups. The relative percentage of CD4^+ ^T-cells within the total T-cells was significantly higher (*P *< 0.05) in both MC-BMT(+) and MC(+) (Figure 3B). Interestingly, the proportion of CD8^+ ^T-cells within the total T-cells was significantly enhanced (*P *< 0.05) both MC-BMT(-) and MC(-) (Figure. 3B). These finding led to significant higher (*P *< 0.05) CD4^+^/CD8^+ ^T-cells ratio in both MC-BMT(+) and MC(+) (Figure. 3B).

### Survival curves comparison

The survival curves comparison stratified based on the histological diagnosis, demonstrated significant (*P *= 0.0014) lower values in the MC group as compared to MC-BMT group (Figure. 4). Additional analysis revealed that MC-BMT (-) and MC (-) had significantly (*P *= 0,009 and *P *= 0,033, respectively) higher survival rates as compared to MC-BMT (+) and MC (+) (Figure. 4). The minimum survival was 25 days post surgery and the maximum survival period was 307 days.

**Figure 5:**
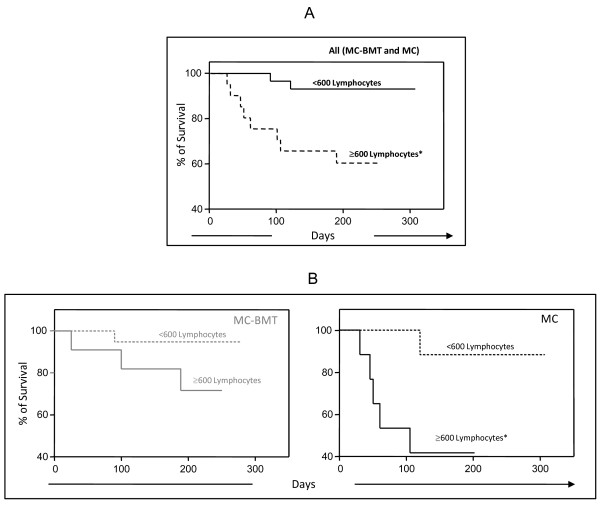
**Survival rates of animals with canine mammary carcinoma**. Kaplan-Meier survival curves for All (MC-BMT and MC) animals (A) categorized according to the lymphocytic infiltrate intensity (<600 and ≥ 600 lymphocytes) further sub grouped according to the histological diagnosis (MC-BMT or MC) (B) (bottom panels). Animals were submitted to quarterly follow-ups during twelve months and survival rates expressed in days between the surgical excisions of the end of follow-up as described in Methods. The survival curves were estimated with the Kaplan-Meier method followed by Log-rank test and significant differences at p < 0.05 highlighted by asterisk.

Additional analysis demonstrated that animals with discrete/moderated tumor lymphocytic infiltrate (<600 lymphocytes) showed significantly higher survival rates as compared to those with intense lymphocytic infiltrate (≥ 600 lymphocytes) (*P *= 0.0035) (Figure 5A). Similar findings were observed in the analysis between MC subgroups (*P *= 0.022), with no differences observed between MC-BMT subgroups (*P *= 0.0871) (Figure 5B).

**Figure 6:**
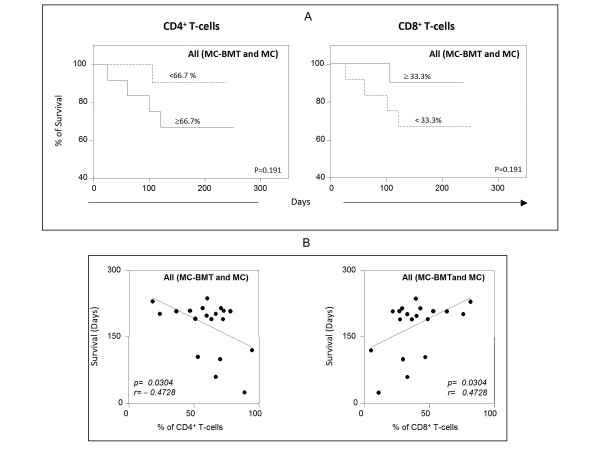
**Survival rates of animals with canine mammary carcinoma**. Kaplan-Meier survival curves for animals for All (MC-BMT and MC) animals categorized according to the relative percentage of CD4^+ ^T-cells (<66.7% or ≥ 66.7%) and CD8^+ ^T-cells (≤ 33.3% or >33.3%) (A). Animals were submitted to quarterly follow-ups during twelve months and survival rates expressed in days between the surgical excisions of the end of follow-up as described in Methods. The survival curves were estimated with the Kaplan-Meier method followed by Log-rank test. Correlation analysis highlighted the significant association between the percentages of CD4^+ ^T-cells and CD8+ T-cells with the animal survival in days (B).

Further analysis demonstrated that animals with tumor infiltrating lymphocytes composition with higher relative percentage of CD4^+ ^T-cells and lower proportion of CD8^+ ^T-cells had lower, but not significant (*P *= 0.191) survival rate (Figure. 6A). Moreover, the correlation analysis revealed that there was a significant (*P *= 0.0304) negative association between the percentages of CD4^+ ^T-cells and the animal survival in days. Moreover, a positive correlation was observed between the frequency of CD8^+ ^T-cells within the tumor infiltrating lymphocytes and the animal survival in days (Figure. 6B).

### Univariate analysis

Histological diagnosis of MC, tumor size > 5 cm, presence of lymph node or pulmonary metastasis, histological grade II-III, multifocal inflammatory distribution, and lymphocytic infiltrate intensity ≥ 600) displayed a negative association with animal survival (Table 2).

**Table 2:** Univariate and multivariate analysis of clinical-pathological parameters associated with low survival rates.

Parameters	Analysis (*P *value)	
	Univariate	Multivariate
**Histological diagnosis (MC)**	0.05*	0.18
**Size (>5 cm)**	0.03*	0.18
**Lymph node metastasis (Yes)**	0.003*	0.09
**Pulmonary metastasis (Yes)**	< 0.0001*	0.39
**Histological grade (II-III)**	0.007*	0.42
**Inflammatory distribution (multifocal)**	0.04*	0.07
**Inflammatory intensity (moderate/intense)**	0.71	ND
**Lymphocytic infiltrate ((600 lymphocytes)**	0.002*	0.02*
**% of CD4^+ ^((66.7%), CD8^+ ^T-cells (<33.3%)**	0.12	ND

* Selected for multivariate analysis. ND = not done. The end point was the animal death or discharge during the follow-up period

### Multivariate analysis

Only lymphocytic infiltrate intensity ≥ 600 (coefficient = 4.1252; SE = 1.8260; OR = 61.8804; confidence interval = 1.7268-2,271.4921; *P *= 0.0239) remained as independent prognostic factor of survival rates in the final model (Table 2).

Additional analysis demonstrated significant association between lymph node metastasis (*P *= 0.04) and clinical stage (*P *= 0.003) with distinct intervals of lymphocytic infiltrate intensity (Table 3).

**Table 3:** Association between clinical-pathological and parameters with distinct intervals of lymphocytic infiltrate intensity

**Parameters**		**Lymphocytic infiltrate**		***p*^#^**
		**<600 lymphocytes**	**(600 lymphocytes**	
			
**Histological Diagnosis**	**MC-BMT**	20	11	0,39
	**MC**	10	10	
				
**Size**	**3-5 cm**	12	06	0,55
	**>5 cm**	18	15	
				
**Lymph node metastasis**	**No**	21	08	0,04*
	**Yes**	9	13	
				
**Clinical stage**	**II-IV**	30	13	0,003*
	**V**	00	08	
				
**Histological grade**	**I**	20	10	0,15
	**II-III**	09	12	

* Significant differences at p < 0.05. ^#^Fisher's exact test.

## Discussion

The functional role of tumor-infiltrating lymphocytes in canine mammary carcinomas is not yet fully established and therefore the analysis of association between host immune response and disease outcome may point out a possible association between immunophenotypic features of infiltrating lymphocytes and their relation to prognostic factors and survival.

In this study, the percentage of T-lymphocytes was significantly greater in the animals from the MC-BMT group without metastasis, suggesting an effective protective role in preventing tumor development. In fast growing tumors, the presence of T-cells constitutes an interesting prognostic indicator when compared with non-immunogenic tumors, since their presence correlates positively with the absence of metastasis in lymph nodes, smaller tumor size, lower histological grade and longer disease free survival time [7].

Additionally, our findings demonstrated that animals from the MC-BMT group with lymph node metastasis presented higher percentage of tumor infiltrating B-lymphocytes. The role of tumor infiltrating B-cells is still controversial. Some studies have demonstrated that chronic activation of B-cells contributes to tumor development by inhibiting the activity of CD4^+ ^T-cells by yet unknown mechanisms [13]. However, our data demonstrated that together with the enhanced levels of B-cells, high frequency of CD4+ T cells was observed in animals with worse prognosis in MC-BMT tumors. Moreover, other studies have pointed out that, the acute activation of B-cells may have a key role in early elimination of neoplastic cells, through the secretion of antigen-specific immunoglobulins, thus participating in the regression of tumors [7,11]. Together, these findings demonstrated that the search for B-cell-related biomarkers to canine mammary carcinoma still needs further investigation to provide a more conclusive hypothesis.

Our data demonstrated that the enhanced proportion of CD4^+ ^T-cells besides the lower relative percentage of CD8^+ ^T-cells leading to higher CD4^+^/CD8^+ ^T-cell ratio observed in animals with metastasis may represent another relevant biomarker to be used in follow-up studies. The maintenance of the CD4^+^/CD8^+ ^T-cell ratio has been pointed out as an important prognostic factor in the progression of human breast cancer [34]. Additional longitudinal studies still need to be conducted to certify this hypothesis.

In fact, the higher infiltration of CD4^+ ^T-cells was observed in the groups with the worst prognosis [MC-BMT(+) and MC(+)], and was associated with tumor progression, metastasis and poor survival. Machetti and colleagues (2006) reported similar results and believe that a greater percentage of CD4^+ ^T-cells is correlated with the induction of angiogenesis, secretion of immuno-inhibitory cytokines and intra-tumor estrogen conversion [35].

On the other hand, regardless the tumor type, the majority of animals displaying increased percentage of CD8^+ ^T-cells did not present metastasis. Therefore, our findings suggest that the number of CD8^+ ^T-cells may represent a good complementary prognosis biomarkers, since there was a significantly higher percentage of this cell population in both groups MC-BMT(-) and MC(-), suggesting that these cells have an inhibitory action, either direct or indirect, in tumor progression that results in increased animal survival. Similar results were observed by Leong and colleagues (2006) [36], assessing the phenotypes of lymphocytes in ductal carcinomas in women. However, Sheu et al. (2008) [34] observed a significant correlation between the increase of CD8^+ ^T-cells and clinical staging in breast cancer. Likewise, Nowark and colleagues (2007) [23] also reported a positive correlation between the number of CD8^+ ^T-lymphocytes located in the tumor stroma and metastasis to lymph nodes in female dogs suffering from mammary carcinoma.

Tumor infiltrating T-lymphocyte may be involved in distinct mechanism depending on the cell subset. It is believed that while CD8^+ ^T-cells play an important protective role controlling the tumor development, the CD4^+ ^T-cells may present a dual role, participating with dichotomic specific immune response to the tumor, controlling or inducing the tumor progression depending on the production of distinct cytokines patterns [8-11,37,38]. Some studies have associated that the predominance of CD4^+ ^T-lymphocytes in the tumor site is capable of "controlling" the expansion of CD8^+ ^T-lymphocytes, thus inhibiting an effective antitumor response [34]. This may explain why some animals survived, despite the high number of tumor infiltrating CD4^+ ^T-cells. It is possible that these CD4^+ ^T-cells may synthesize pro-inflammatory cytokines related to the protective anti-tumor immune response. It has been demonstrated that the presence of tumor-infiltrating lymphocytes in breast carcinomas associated with the production of potentially anti-inflamatory cytokines, such as IL-4, IL-10, and TGF-β would be associated with an immunomodulatory microenvironment, triggering mechanism of tumor escape from the immune system [17].

It is possible that the activation of both pro-inflamatory CD4^+ ^T-cells and cytotoxic CD8^+ ^T-lymphocytes in adequate proportions leads to an efficient immune response that control the growth of tumor cells [39]. However, the role of pro-inflamatory CD4+ T-cells in the tumor control is still controversial.

The presence of inflammatory cells in human tumors has been linked by some researchers to an immune response inefficient in preventing tumor development [16,35,40]. There is evidence that some inflammatory cytokines (such as IL-1β, IL-6, IL-23 and TNF-α promote tumor development by acting directly or indirectly on neoplastic cells [10,15,16]. It is possible that the balance between pro- and anti-inflamatory cytokines is in fact more relevant than the isolate analysis of a given cytokine.

In our study, several clinic-pathological parameters showed significant association with the survival rates, including the histological type (MC), size (>5 cm), the presence of lymph node and pulmonary metastasis, the histological grade (II-III), the inflammatory distribution (multifocal) and the lymphocytic infiltrate (≥ 600 lymphocytes). These findings re-emphasize previous reports that highlight the importance of monitoring these clinic-pathological features in order to understand the biological behavior of mammary cancer [41-48]. Although, these parameters provide valuable information regarding diagnosis, prognosis and therapy [46,47], the lymphocytic infiltrate intensity (>600) was the only prognostic factor with independent association with lower survival rates in our investigation.

The higher numbers of infiltrating lymphocytes, along with the presence of metastasis were observed in the groups of animals with lower survival rates. As the survival in days displayed a negative association with the % of CD4^+ ^T-cells and positive correlation with the % of CD8^+ ^T-cells, it is possible that the higher number of infiltrating lymphocytes may reflected in higher number of CD4^+ ^T-cells and together these variables may be biomarkers related to worse prognosis. On the other hand, animals with lower of infiltrating lymphocytes, but those with preferentially infiltration of CD8+ T-cells may present better prognosis.

## Conclusion

Our data demonstrated that from the immunophenotypic standing point, the analysis of tumor infiltrating CD4^+ ^and CD8^+ ^T-cells may represent important complementary prognosis biomarkers to be further investigated in canine mammary carcinomas. The higher percentage of infiltrating CD4^+ ^T-cells was observed in animals with the worse prognosis, including those with lymph node metastasis and lower survival in days. Moreover, the percentage of infiltrating CD8^+ ^T-cells was correlated with absence of metastasis and higher survival span in canine mammary carcinomas. The importance of B-cells as a prognostic biomarker still remain to be elucidated, may be approaching further analysis of B-cell subsets and their activation status.

Furthermore, additional studies are still needed to expand our understanding of the complex mechanisms involved in the function and interaction of infiltrating CD4^+ ^T-lymphocytes, their cytokine biosynthesis, as they relate to increased malignancy and metastasis, and of the role played by CD8^+ ^T-cells in inhibiting tumor development, determining the tumor microenvironment that favor the control mechanisms in canine mammary carcinomas.

## Abbreviations

**MC-BMT**: Mammary carcinoma in mixed tumors; **MC**: Mammary carcinoma; **(+)**: presence of metastasis; **(-)**: absence of metastasis

## Competing interests

The authors declare that they have no competing interests.

## Authors' contributions

**AEL**: conceived the study, participated in the immunoassays, performed the statistical analysis and drafted the manuscript. **MSSA**: carried out the immunoassays, performed the statistical analysis and participated in the design of the study. **JMCN**: carried out the immunoassays and participated in the design of the study. **SMBM**: Analyzed and interpreted clinical date. **ATC**: participated in the design of the study. **SVC**: performed the statistical analysis and interpreted and analyzed data. **OAMF**: participated in design of the study and interpreted and analyzed of data cytometry. **RS**: participated in the study design and revised the manuscript. **GDC**: participated in design and coordination of the study and revised the manuscript. All authors read and approved the final manuscript.

## Pre-publication history

The pre-publication history for this paper can be accessed here:

http://www.biomedcentral.com/1471-2407/10/256/prepub
